# Report on Practical Dentistry, at the Eighth Annual Meeting of the Am. Soc. of Dental Surgeons

**Published:** 1848-10

**Authors:** C. O. Cone

**Affiliations:** Baltimore.


### *Plugging the Organ to the end of the Fang.*

This course is generally adopted after a change has occurred in that part of the nervous pulp nearest the dental decay, or of the whole of the same, from chronic inflammation; or the collateral indications for the capping the nerve, by any of the methods named, would argue an unsuccessful termination. The late Dr. Edward Hudson, of Philadelphia, was, however, in the constant practice of treating the nervous pulp of all the anterior teeth, and even the bicuspides, when, from the depth of the decay, the unavoidable exposure of the nervous pulp was made necessary, in the following manner: He proceeded to destroy the nervous pulp in part, or entire, by running a small instrument up the natural canal of the tooth, as high as where the nervous pulp passes through the foramen at the end of the fang. The instrument there meeting with resistance from the dininished size of the canal, cuts off the nerve at that point. The remnant of the destroyed tissue is to be removed,* and after sufficient time has been afforded for the discharge of a small quantity of serum, which usually takes place from the wounded portion and its cicatrization, the fang and cavity is ready to be treated in the manner to be described.

This method of treating the nervous pulp when exposed, was proposed to be applied in similar cases, in molar teeth, in a paper read before this association by Dr. E. J. Dunning.† But the report would express a doubt in relation to the permanent success of this practice. The external periosteum, or *perodental* membrane of the molar teeth, or multiplied-fanged teeth, are less highly organized, and the nutritive vessels which supply the teeth from this source are fewer in number, and smaller in volume, than in teeth having but one fang, and occupying the anterior portion of the arch. The physiological law, that no organ can sustain a double duty for a length of time, without being marked by, or yielding to disease, is early developed in

* American Journal of Dental Science, vol. i, p. 171.

† Ibid., vol. vi, p. 15.

the pero-dental membrane of the molar teeth, when the nervous pulp has suffered destruction; and the nutrition of the organ follows the envelop of the fang, which at first becomes partially and then wholly destroyed, and the extent of the necrosis of the fang of the tooth corresponds to the progress of the destruction of membrane last named. This is not unlike the history of the anterior teeth, which have been subjected to this species of operation; only the destructive process is more slow and tardy in its course, for reasons before given; and the fangs more subject to exostosis and other diseases dependent on slow but continued periosteal irritation.

Your report is aware of the argument, which many members will feel capable of urging against the position which the report assumes at this point; by assuring the association that they have seen teeth, treated in the manner above described, remaining in the mouths of patients for years, without producing marked symptoms of disease. This, your report will grant, without feeling that the justice of the report, or the truth of its observations has been assailed. But when such success as just named attends the operation, it is when the patient, and organ operated on, offers the greatest amount of guarantee for its success, by the nerve not having been the subject of previous inflammation; the patient in vigorous health, and with a good constitution, and in age about the "prime of life." But such arguers of success and results are few, compared with the whole number of cases which are attempted. And even when the greatest amount of success attends this operation, which its friends would argue to maintain, a long time never elapses without a partial or entire *necrosis* of the fangs of the teeth thus treated; and if it is not the agent of vexatious trouble to the patient, to the pathological eye, venous injection of the soft parts, and other structural changes of the surrounding tissues, can always be detected; and the true and only just method of meeting the argument of the report by objectors, is to exhibit teeth that have been thus treated for a period, removed from the mouth, without exhibiting any morbid appearance.

Some operators, being impressed with the above truths, have endeavored to obviate these objections, by excising the nervous pulp with a sharp pointed cutting instrument, at the neck of the tooth, leaving that portion which occupies the fang to supply the same with nutritient vessels. When inflammation of a high character is not attendant on the excision of the nervous pulp at this point, (which is not seldom,) the operation is accomplished without violating the physiological and pathological laws of the system, which the total destruction of the nervous pulp in the crown and fang of the tooth incurs. To insure a successful attempt in this operation, all the indications for its performance should be of the most favorable character.

After an operation like the one last described, it is not unusual for the remnant of the dental pulp, as sometimes happens after amputations, to become very large, and the sensibility morbidly increased;* or the development of a fungous growth, agreeably to Dr. S. P. Hullihen's description of this disease,† which fills the vacancy in the crown of the tooth, and presses against the plug and defeats the operation.

From the pain and terror which accompanies this method of destroying and excising of the nervous pulp, was suggested the use of some escharotic, combined with some narcotic agent, and which has been employed by the profession to a great extent, for effecting the disorganization of this tissue, previous to its removal from the canal of the tooth, preparatory to the operation of plugging. There has been a variety of caustics used for the purpose named; but the two most frequently employed are chloride of zinc, which has been more extensively used in England than in this country. It possesses the quality of destroying tissue equal in bulk to the caustic used, which is not the case with the other agent used for this purpose, namely arsenious acid, which is well known in general surgery as offering a demand to be used with great caution, not only from the irritation it may give rise to, not only in the immediate vicinity of its application, but to the health of the general system. These objections, however, assume a less serious aspect, when used

* Gross' Pathological Anatomy, Boston ed., 1839, vol. i, p. 481.

† Western Journal of Medical and Physical Sciences, No. 35, p. 558.

as an escharotic for the purpose under consideration, as its effect on the general system, through the medium of absorption, could hardly assume anything like an alarming effect, and much less fatal, from the small quantity of this agent demanded for the operation, unless the patient's system presented a peculiar idiosyncrasy of susceptibility to the irritation of this agent.

The mode of application of this material is so familiar to the profession, that it would be unnecessary for a description to be given; but a few remarks in relation to its action, is not thought inappropriate. When administered in a proper quantity, and the absorbents do not spread its action, after a free contact, the nervous pulp will be found disorganized, and its structure infiltrated with serum; and although the touch of an instrument may give pain, when forced through the foramen which gives access to the pulp; by enlarging the orifice of this foramen, that the introduction of the instrument shall not exert pressure on the parts at the superior portion of the fang, where the nerve is still in an inflamed state, but not disorganized; it may be removed with little or no suffering to the patient. After a lapse of a short time, for the inflammation to subside in that portion of the nervous pulp nearest the superior extremity of the fang, without the confinement and consequent pressure of a serous fluid, which most frequently takes place, to a greater or less degree, from the remaining organized part of the nervous pulp; the next step in the operation is prepared to be taken.

It is evident to your report, that the use of this agent for the purpose under consideration, has been productive of serious evil, from its injudicious administration—not varying the quantity in accordance with the indications of the case, or entirely withholding its use, when the physical indications would pronounce such an edict. The practice of dental surgery demands the members of the profession, to subject every case to which they are called to dispense the benefits of their profession, to a careful investigation, in which the only witnesses admitted, to the total exclusion of prejudice and preference, should be science and facts, and the umpire correct judgment, to which the remedial agents should bend submission under all circumstances.

An over dose, or improper administration of arsenious acid, is marked frequently by the injection of the tubular structure of the dentine, with the fluid portion stained with the coloring particles of the blood, which at first gives the crown of the tooth a faint pink complexion, but after a short time, a chemical change is produced by the light and air, on the coloring particles of the injection, and it assumes a permanent purple hue.

This result is most frequently seen in young patients, whose tissues are highly vascular, or whose system has been enervated by disease or injury, and most plainly made visible in teeth of a delicate structure, and highly organized, although teeth of all degrees of density and complexion are capable of exhibiting this appearance. If absorption has taken place, from the quantity of the agent used, or susceptibility of the system to the influence of this agent, the tooth will develop pain when pressed, or comes in contact with any substance, and appears slightly elongated from inflammation existing in the *pero-dental* membrane; and the tissues surrounding the fang will present a greater or less degree of capillary injection.

It is not unusual, when absorption has occurred, for the same, and the inflammation upon which the latter depends, to affect only a small portion of the pero-dental membrane, as is shown by the tooth not exhibiting tenderness when pressed with force, except in one direction. The inflammation in these cases may assume an active character, attended with great pain, and of that order which marks periosteal inflammation; and which is greatly aggravated by the inhalation of cold air, or the introduction into the mouth of anything that shall change its temperature. Thus, the inflammation may run its course, and if the tooth be not extracted, its fang left in a partially or wholly *necrosed* state, according to the extent which the pero-dental membrane has been implicated in the inflammation. The inflammation may, instead of assuming the character described, resolve itself into a chronic, or sub-acute state, subject to change from this to acute, and back again, when an irritant makes an impression on the system, which is felt at this point; giving rise to exostosis, and other troubles dependent on slow and changing periosteal inflammation; but eventually loses its vitality, and the tooth its vessels of nutrition.

The absorption, and attendant unfortunate results, is most frequently exhibited in young subjects, and those whose constitution indicates a predisposition to inflammation, and is lessened as the patient's health is firm, and constitution good, and his age near or past the meridian of life, when the absorbents have lost the activity of youth, and the vascularity of the tissues lessened. From a want of regard for these, and other indications and cautions, and the reckless manner with which arsenious acid is generally used, and its injudicious application, a large amount of injury is done to patients and the profession; and the success, which has been greater in the hands of Dr. E. Maynard, of Washington City, is to be attributed to the discrimination and prudence which marks its use in his hands, and the completeness of the subsequent steps of the operation.

After the canal of the tooth has been prepared and excavated to the superior extremity of the fang, and the cavity prepared for the reception of the gold; heavy numbers of foil should be used, by being prepared so as to enter the canal on the point of an elastic plugger, with slight irregular projections presenting themselves from the side of the instrument, for the purpose of conveying the gold up the cavity of the fang of the tooth; condensing it each time a fold is carried with the instrument to its place; thus continuing until the pulp cavity is filled into the crown of the tooth, when the operation is to be finished in the usual way.

That the means above proposed for the destruction of the nervous pulp of a tooth, and then plugging the fang to its end, has many important practical bearings, cannot be questioned. In the first place, it is a resort when all other means are forbid by the peculiarity of the case, and when judiciously resorted to, agreeably to indications, is an important instrument in dispensing the benefits of the profession. And while the profession can hold this self-congratulation, at the same time it must be acknowledged that weighty objections can be offerred against its general and indiscriminate practice.

The plugging of the fang to its apex, for the purpose of preventing pain in the organ, from the irritating fluids accumulating in the canal, and destroying the parietes of the same, and setting up inflammation in this, the higher organized structure of the tooth, causes these fluids, which cannot find egress through the canal of the tooth, to inflame the already irritable structure about the apex of the fang; and the peri-dental membrane, at its superior extremity pours out plastic lymph, which become organized and condensed into a sac, in the centre of which pus is secreted, and the sac in fact forms the coat of an abscess. The quantity of pus is usually small, and varies in quantity according to the extent which the parts depart from health; but it is always sufficient to exert pressure on the investing parts, and induce ulcerative absorption, by which the pus gradually makes an outlet through the gum, generally opposite the end of the fang of the tooth thus circumstanced.

The formation of an alveolar abscess, having like results, may form from blood being poured from the end of the arterial vessel of an excised nervous pulp, or when an escharotic has been employed; which at first is of a dark complexion, and of a fluid consistency; but after a short time the surrounding absorbents remove the coloring particles, and the serous portions of the blood, which consequently become not only lighter in complexion, but more dense and firm. At still a later period, the clot becomes organized, and frequently encysted,* and secretes pus, which is poured into the mouth through a communication from the same to the surface of the gum, opposite the fang of the tooth.

The above state of things may exist with the organ, the superior portion of the fang in a *necrosed* state; while that portion of the fang nearer the neck of the tooth, has its vitality supported by the peri-dental membrane, which has not lost its vitality; but the function and health of which is to be gradually encroached on by the morbid influences at its superior ex-

* Gross' Pathological Anatomy; Boston ed., vol. i, p. 83.

tremity, until pus may be forced from some point between the gum and neck of the tooth, which is followed by, or attended with an increased vascularity, and thickening of the tissues surrounding the fang of the tooth, which becomes partially or wholly *necrosed*, from the end of the fang to the neck of the tooth; and when the tooth is extracted, its fang is found streaked with pus, and a rough surface marking the same, and at the apex of the fang it appears as if worm-eaten, and at other times showing a deposit of a greater or less quantity of *cementum*, at various points of location on the fang, according to the character and progress of the inflammation; but usually the deposit is greater nearest the end of the fang, where it may have been subject to chronic inflammation for the greatest length of time; which inflammation is more frequently followed, in the periosteal tissue, by thickening and a deposit of osseous matter.*

Your report would not be understood as saying, that the above described results are always the immediate attendant on this course of practice; but that its progress of visitation is according to the judicious selection of cases, and the ability and care with which the operations are conducted. But that the result finally is such as above described, if sufficient time is afforded to elapse, it is believed to have been established.

It now becomes necessary, in this connection, to speak of two agents that have been introduced recently in the operation of plugging teeth. The first to be named is, *tissue paper;* the superiority of this article over all other agents previously used for drying a cavity in a tooth, previous to the introduction of foil, (a consideration of no small importance,) cannot be questioned; and your report would recommend the members of this association to test the truth of the above, by instituting a trial of this agent in their operations.

The next, is a compound, known as "Hill's stoppings," or "soft stoppings," &c., which has been introduced into the profession as a materal for plugging decayed teeth; but which can hardly be brought into extensive use by the physiological and

* Gross' Pathological Anatomy; Boston ed., 1839, vol. i, p. 350.

pathological dentist. The most available argument for its use is, that it can be used where no other material can, and is a substitute for amalgam, as it is capable of fulfilling all the merits claimed for that agent by its friends, without the same objections being urged against its use. This new compound, it is true, is not subject to the same number of objections for filling or plugging teeth, which amalgam is, from the direct injurious effect of the latter; but is equally vulnerable to the argument of its producing indirect injury; as it is claimed to retain certain teeth in the mouth, which cannot be preserved by plugs formed of gold foil, when it is generally urged, by those who hold the highest stations in the profession, that any dental organ that can be *preserved* without injury to the surrounding parts, can be plugged with gold foil. This agent proposes to retain in the mouth, organs which cannot be preserved with gold, and which must consequently have assumed some one of the unhealthy pathological changes, previously described in this paper, the indications of which argue extraction instead of efforts towards preservation, and that necessarily at the expense of the the health of the surrounding parts. And until some substance can be found, against which similar and other objections cannot be urged, the search of the alchemist for that which shall turn the baser materials to gold, for dental purposes, is not at an end.*

From the neglect of the employment of the preventive means in the hands of the skilful dental practitioner, or from the unskilful application of these means to the demand of the case; and perhaps notwithstanding all care and vigilance to prevent the progress of dental disease; the removal of the tooth becomes necessary, to subdue the suffering of the patient, or limit the extent of the injurious impression of the diseased organ; and the best means for the safest and effectual execution of the ope-

* It may be proper to observe, in justice to Dr. A. Hill, that his experiments in relation to this article are yet incomplete; and at the same time, would refer the reader to an article in another part of this Journal from Dr. Hill, in which he endeavors to correct what he esteems errors or false impressions inculcated by so much of the report as refers to "Hill's stoppings."

ration, is an important and anxious inquiry with the reflecting operator, previous to proceeding to the

### *Extraction of Teeth.*

It is not in accordance with the design of the report, to give explicit and definite directions for every specified case that might occur in practice, but to confine the remarks and the attention of the association to general principles and considerations, connected with the subject; with an occasional deviation from this prescribed course, for the purpose of noticing the introduction of improvements in this department of the profession.

For the faithful execution of this operation, it is an indispensable requisite that the operator be familiar with the anatomy of the organs and the surrounding parts, on which he may be called to exercise the functions of his profession in this department of his duties. Any person who undertakes the extraction of the teeth, should be conversant with the manner in which they are placed in their sockets. An accurate knowledge of the parts gives confidence; for, however greatly the form and position of the teeth may differ in individual cases, the leading features of the arrangement, and anatomical developments are generally the same.

To fulfil the obligations of the operator, and extract teeth *skilfully*, confidence in his own practical abilities, is an indispensable qualification. On examining a tooth to be removed, the operator should feel full confidence of success, and take every means to ensure this end; and if he has any doubts of the success of his ability, the arguers of a desirable result are much lessened; and whatever the operator determines it expedient to do, must be accomplished with unbending firmness, permitting nothing to surprise him in the discharge of his duty, which shall produce vacillation of judgment, but be prepared for every accident and disappointment that may throw itself in the way to defeat the purpose, as if waiting in expectation of such, ready to overcome all impediments, by a ready applicability of means to the end, with unflinching determination and confidence.

To secure the ends above named, the operator demands a familiar knowledge of the pathology of dental and buccal disease, to enable him to determine the amount and character of opposition or facility which they may offer in the performance of the proposed operation; and the physical indications, which can alone form the means of determining the diagnosis of the case, and establish, before an effort is made for the removal of the organ, the force with which the tooth will resist the separation of its particles from the surrounding socket; that the most effectual means may be adopted and chosen, to overcome, with facility and safety, the strength of opposition which may be offered.

When the operator has attained the above qualities, he is prepared to institute inquiries in relation to the merits of different extracting instruments.

The instrument, if not most frequently used by the profession, is more generally employed, if we number *all* that succeed in the dislocation of a tooth from its socket, is the key instrument of Garengeot, which may be properly considered, in mechanical language, acting as a wheel and axle; and when used, the hand of the operator applied to two spokes of the wheel, acts as the motive power, while the tooth is fixed to the axle by the claw, and is drawn out, or, more properly, obliquely pressed over or upset, as the axle turns. The gums and their osseous base forms the support on which the axle rolls.*

There are some serious objections to the use of this instrument, from its action and inapplicability to the variations which attend different cases in which it is employed. For the removal of a tooth, without doing injury to the socket in which it is held, the force applied for its removal must be either in a perpendicular or oblique direction. The former is precluded in practice with the instrument under consideration, and the latter rarely ever attained; as the claw in turning, describes the segment of a circle much too small for the tooth to be raised in an oblique direction; and the moment pressure is applied to

*Arnott's Elements of Physics; Philadelphia ed., 1831; vol. i, p. 442.

the shaft, the axle turns abruptly on its support, and drags the claw in a parallel direction, the effect of which is to crush down the alveolar process, and cant out the tooth, the claw of the key preventing the tooth from rising over the edge of the socket; or else the tooth is fractured at its neck, from the parts not yielding to the horizontal power, or a complete failure of the operation, from inability to move it in its position.

The difficulty of determining the space required between the edge of the claw and the fulcrum of the key instrument, is a difficulty that often embarrasses the operator; for, if the space in the least exceeds the width of the tooth, as soon as the instrument is brought in action, the fulcrum will not act in unison with the claw, but will change its position most rapidly, subjecting the instrument to loss of power, and the risk of actual failure proportionally increased. In like manner, if the space should not prove sufficiently wide, the claw will not permit a horizontal line to be drawn with the fulcrum, and the effect will be the same as if the latter had passed below its proper position.* This difficulty is often increased by the soft tissues being inflamed, which retracts or permits the space to diminish when necessary pressure is applied to the fulcrum in the removal of the tooth.

For reasons similar to those enumerated, and others, the *majority of the profession* use the forceps for the operation of extracting the teeth. This instrument may be said to be of modern introduction into the profession, for the extraction of *all classes* of teeth, and when having a firm articulation.

The utility of this instrument in the hands of a skilful practitioner, rests much on the manufacture of the same. If the beaks or mouths of the blades be badly adapted to the necks of the teeth for which they are constructed, the success of the operation is much lessened. This fact was felt by Mr. Snell of London, who first made any considerable improvement in this feature of the instrument, and so adapted the beaks of the forceps to the neck of the tooth, in a manner that the instru-

* Clendon on Extraction of Teeth; London ed., 1843; p. 10.

ment might be inserted low upon the same, and often to the bifurcation of its fangs, the cavity formed by which, is designed to be occupied by a corresponding point or projection from the centre of the extreme edge of the beak, making the lips of each of the same describe the shape of two small crescent figures; when made to be adapted to the inferior molar teeth, and correspondingly, more or less, as the instrument is made for the various classes of teeth; thus lessening the arm of support, the depth by which the tooth, in a great measure, is sustained with firmness.*

Still later, Dr. J. F. Flagg, of Boston, made an improvement in the shape, &c., of the forceps, by adapting the hawk's-billed forceps more accurately to the form of the tooth at its neck, and giving the handles a shape and position more natural to the operator, and enabling him to command and apply to the instrument a larger amount of physical force when demanded. This end, however, was attained to a greater degree by Prof. C. A. Harris, of Baltimore, who adapted to the beaks of Mr. Snell's forceps, handles which not only increased the power of the instrument, by placing the motive power, or the hand, nearer on a line with the long axis of the tooth; but the shape of the handles was changed, by so placing the angles and curves of the same, as to bring the hand nearer the chest of the operator, and placing the muscular portion of the hand of the operator beneath the instrument, giving the operator, by this means, a greater command over it, without the loss of physical power. The design of these instruments is often violated by faulty construction, and in one particular almost universally so. The palatine fang of the superior molar does not occupy a medium position between the two external fangs, but slightly posterior to the position named; an anatomical feature scarcely ever observed by the manufacturers of forceps, which gives the instrument, when faulty in this particular, occasion to adapt itself badly on the palatine side of the tooth, and often giving the organ a slight rotary motion, which, during

* **Müller's Physics and Meteorology, Philadelphia ed., 1848, p. 75.**

extraction, much oftener fractures one or more of the points of the fangs of the molar teeth, than frees them from the alveolus uninjured in form and shape.

The difficulty experienced by operators in introducing the ordinary shaped beaked forceps, high upon the fang of the superior molares, from the abrupt divergence of their fangs, and the difficulty consequently experienced in extracting these teeth, when decay has extended near or to the bifurcation of the two external fangs, caused Dr. E. Maynard, of Washington City, to introduce a hook on the outer or external beak—occupying the centre of the same—of the ordinary forceps, for the extraction of that class of teeth; for the purpose of penetrating between the two external fangs.

The utility of these forceps, *as shown in a plate accompanying the American Journal*,* can be much improved by constructing the hook above named on the beak of a forceps having its usual breadth or crescent shape, extending the hook from the centre of the same, about the sixteenth of an inch upwards, before giving it its curve towards the opposite beak; and, instead of a rounded form, let the hook be flattened, that it may penetrate between, without fracturing the external fangs of the tooth, at the point of their bifurcation.

The success of an operation performed by the assistance of the forceps, depends much on the manual dexterity which the operator has gained in the use of the instrument; and the command which marks its use in his hands during an operation. The forceps must be accurately adapted to the neck of the tooth, and pressed as far down on the fang of the same as it is capable of being placed, before an effort is made to detach the tooth from its socket; and if during extraction, the organ refuses to yield on one side, the instrument should be so used, as to apply the extracting force in that direction which yields with obstinacy. The success, as well as the pain of the operation, will be lessened, if the operator executes his duty with firmness, and a calm determination of accomplishing the end proposed.

* American Journal of Dental Science, vol. vii, p. 289.

In this connection, your report would refer to the introduction, in the practice of the profession, of *sulphuric ether*, *chloroform*, and a similar agent, known as *aldéhyde*, claiming some superior merits, according to French authority,* over the two first named, as an anæsthetic agent, for the purpose of subduing pain and sensibility during the extraction of teeth, and the performance of other surgical operations.

Your report does not propose to enter into an analysis of the properties and effects of these agents on the human system, as these points have all been more fully and ably discussed in other places, than the limits of this report will permit; and the observations offered will be chiefly confined to the indiscriminate use of those agents by the profession generally, and the injurious effect to the same, from the objectionable manner in which they have been introduced to the public, instead of professional patronage.

No sooner was it known that an anæsthetic agent existed, which could be exclusively secured to individual members of the profession by *letters patent*,† than a host of men rushed to purchase superiority, which education and talents forbid, and indolence and qualities denied; and the eye of the honorable dental surgeon was dimmed by honest indignation, at the injury done the profession, as his gaze met in the public prints, fulsome advertisements, headed, "Dentistry without pain;" "Now is the time for artificial teeth;" "To the toothless;" "More chloroform;" and various other advertisements of like character, any one of which would have disgraced the estimation of any calling, in the opinion of the honorable and philosophical mind. It is true, that these charges of disgraceful and unprofessional conduct, are not applicable to the regular practitioners, except in a few instances. Nevertheless, the impression effected on the profession, in many instances, was not the less injurious, as the distinction between the practitioner who adminis-

*Archives Génerales de Médecins, tome xvi, p. 525.

† It may be proper to remark, lest a false impression be made, that Dr. Morton has, with commendable magnanimity, annulled, so far as he is interested, the patent above referred to.

ters an anæsthetic agent, as well as dispenses all branches of the profession mechanically, is not so easily distinguished by the undoctrinated, from the scientific and well educated dentist, who, instead of self-laudation, modestly relies on his conscious merits of fulfilling the professional demands falling to the dental surgeon.

No question of argument is offered in relation to the merits of these anæsthetic agents, in producing insensibility of the nervous system; as all that its friends reasonably demand in this particular will be granted, it being only an assertion, in another form, of the power of these agents, and of the discriminating caution which should attend their administration.

But the above fact seems to have never been known, or to have been forgotten by a certain portion of the profession, who know no discrimination in their use, but a request for their administration; while others, more cautious, have failed to impress the importance of this feature; and hence it has been used for the most trifling surgical operations in the profession, by those incapable of exercising discrimination; without doubt to the serious injury of the patients often; as the class of operators to which reference is made, have not the most remote idea of the difference between the sound of a healthy lung and a diseased one, and have not the slightest conception of the means of detecting any one of the diseases of the heart, yet boldly administer the dangerous narcotics known as ether, chloroform and *aldéhyde.*

To the above, many of this class of practitioners would make issue, by assuring the public and the profession, of the harmless powers of these agents. To this and like assertions and arguments, your report would reply, that if the administration of these anæsthetic agents can do no harm, it is utterly impossible for them to do any good. This is as perfect an axiom as any one of those that sustain geometry. An equally strong argument in support of the dangerous power of these agents, from their indiscriminate administration, is found in the fact that hardly a medical journal comes to hand without containing an account of some new remedy for counteracting the evil effects of these agents; and what fact could be more expressive of their power and danger? If mercury did not salivate, we should not hear of remedies for *ptyalism*. If arsenic, opium and other poisons did not injure the system, we should not hear of antidotes and counteracting agents. If the medical maxim be received and adopted, that in proportion to the difficulty of curing a disease is the number of remedies increased; we have abundant proof of the stubborn and dangerous effects of anæsthetic agents on the human system.

Another class of dentists more than admits all that is urged above, but still know no caution, nor have the qualities for distinguishing the observance of the same; make free use of the article, and under all circumstances, and then make a placard or walking advertisement of the victims of their practices, to puff up, not so much the anæsthetic agents, as the wonderful genius who uses it, and who, "thank God, is not as other men."

Mystery is a great distorter of truth, and it may be a subject of regret that it should anywhere exist. But with such men as named above, it is the idol they most worship. It is the jackal that feeds the sordid cupidity of quacks, from the forest of public credulity. The hurrah in the public belief of the virtues of a chemico-dental discovery, which possesses magic power; or the wonderful element of a secret instrument, or the efficacy of a new and peculiar method of inserting artificial teeth; the product of his laboratory has gone through the country, and died away. The hum through the community, of the relationship to a favorably known member of the profession, from a similarity of name, has ceased to bring patients. An unsolicited call upon a member of the profession, held in high estimation by the same and the public, can no longer be tortured into a period of studentship. A partial course of lectures, or a failure to sustain a final examination, is no longer known in the eye of the community, as an honorable and honestly acquired diploma from a respectable dental institution. The curtain has fallen on the darkest of his duplicities; the offered professorship in some dental college, the first principles of which are taught in the same, his mind is incapable of comprehending; or an honorary title from an institution, at which his morals are well known not to answer the test for such a consideration, as his qualities are deficient as a practitioner, in comparison with the lowest of its students. But here comes the rescue of his tottering hope and fortune: a remedy, in the hands of every one else powerful and dangerous, but in his, a *safe*, *efficient*, *agreeable and heavenly transporting agent.*

Thus every mountebank, who digs out a corn, and dignifies himself with the title of chiropodist; every itinerating dentist, who gouges out a tooth or fills a cavity with amalgam; or any thing that can creep, or crawl, or sneak into any of the unguarded sanctuaries of medicine, can arm himself with an inhaling apparatus, and a bottle of anæsthetic material, with which he expects to prey on the public; and the rush to his rooms, by credulous patients, to wait their opportunity for the indiscriminate, or more properly mechanical administration of these narcotic agents, would not suffer in comparison with the attractions of a *circus* in a country town, or the crowded infirmary of an itinerating lecturer and "curer of consumption;" and if it was not for the disgrace to the profession, and danger to the patients—the gravity with which they announce that the use of these agents, in improper hands is dangerous, but in theirs perfectly safe—it would be most amusing.

Your report desires it to be distinctly understood that the point of discussion rests on the abuse of anæsthetic agents, and the consequent disgrace brought on the profession generally, and the danger and injury to patients from their indiscriminate use; and the report would urge the seal of disapprobation to be placed on such practice by this association; that the honorable portion of the profession shall not suffer with those guilty of indiscretion, to call it by no harsher name.

The value of sulphuric ether and chloroform, as a narcotic and a therapeutical agent, can hardly be estimated. They are equal in value and power, in the medical catalogue, with *strychnine*, *morphine*, *iodine*, and Fowler's solution of arsenic; but to popularize these agents, and urge or even admit their general and indiscriminate use, would be the opening of a new Pandora's box on the world; and it must be honestly confessed by the well educated, that as little harm would result from popularizing these articles, and their indiscriminate use, as of ether or chloroform.

In conclusion, your report is of opinion, that the greatest, and as yet unanswered objection to the employment of anæsthetic agents, is its narcotic influence, and one that applies with equal force against its indiscriminate application, and even its employment at all, except in very painful surgical operations, the severity of which will justify the positive and direct interference with the functions of so important and delicate a structure as the brain, either of which the anæsthetic agents plainly produce. Hence, in all minor operations in surgery, their administration is forbidden; and that their demand in the practice of dental surgery is small, and in such instances is confined to cases which require a familiar knowledge with the functions of the human system during health and disease, for their safe administration.

### *Hemorrhage.*

Hemorrhage, of more or less severity, is a frequent and often a troublesome attendant on the extraction of a tooth, which may be dependent on a hemorrhagic diathesis, or some irregularity in the constitutional functions of the system, whereby the vessels are in a morbid state of activity;* and the blood is suffered to escape, which should have been suppressed by a simple process of nature, when the system is subject to the laws of health; or, as is more frequently the case, from the dental artery which is retained by its attachment to its alveolus not being ruptured, during extraction from the same, below the point at which it leaves the bony canal to enter the foramen at the apex of the fang, and thus prevented from contracting on itself and suppressing the flow of blood from the mouth of the artery, agreeably to the laws of nature.

* Gross' Pathological Anatomy, Boston ed. 1839, vol. i, p. 78.

For the purpose of subduing hemorrhages under the circumstances above named, a variety of constitutional and local remedies are familiar to the profession. But during the last year, two substances, before unknown in this relation, have been brought to the notice of the profession, for the purpose under consideration. The first that will be named, was brought to notice by Dr. B. B. Brown, of St. Louis. The article referred to, consists of worn linen, cut in strips of such width, that when rolled upon itself, slightly oblique to the length of the strip, having previously received a coating of melted beeswax, it shall take the shape of the socket of the fang of the tooth extracted, and by its pressure, exerted on the mouth of the dental artery, or other bleeding vessels, suppress the hemorrhage. When the tooth extracted has more than one fang, and the hemorrhage proceeds from the socket of each fang, the multiplication of these impermeable compresses becomes necessary.

The other agent referred to for controlling hemorrhage, is a solution of *gutta percha*, the properties of which, in this relation, were communicated to your reporter, in a communication from G. F. J. Colburn, dentist, Newark, N. J., in answer to the appeal made by the chairman of the committee, to the dental profession generally, for information, &c., published in the American Journal of Dental Science.

He says, "Last winter, soon after the introduction of chloroform into this country, *I discovered* that it would, with a small addition of oil of turpentine, readily dissolve *gutta percha*, producing a solution possessing considerable adhesive properties, and which becomes solid after a few minutes exposure to the air, and is impervious to moisture, and uninjured by exposure to the atmosphere.

"I found that this solution would cement the broken parts of plaster casts so firmly in a few minutes, that it was found almost impossible to separate the pieces; so, also, when applied to any solid porous body. This fact, together with a knowledge of its properties in withstanding the action of moisture and acids, the idea suggested itself, of applying the solution to a useful purpose in pivoting artificial crowns of teeth on natural fangs, for the purpose of protecting the fang at the junction of the two, from the moisture, aliments and secretions of the mouth. I have used the preparation in numerous instances, for the purpose named, and, as far as I have ascertained, with beneficial results.

"This preparation is valuable for uniting wounds by first intention, and for suppressing hemorrhage, when applied to the wounded parts. I have tested its properties, in this relation, by applying it to the gum, when the decay of the tooth made it necessary to wound the former in the preparation of the cavity, and in suppressing hemorrhage following the extraction of teeth. I would therefore offer it to the profession as a valuable *cement*, an *impervious coating for pivoting teeth*, and *as a styptic.*

"I send you some of the *gutta percha*, which you can dissolve by placing in a bottle, and adding to the same four parts of chloroform to one of oil of turpentine. The bottle should be kept tightly corked, to prevent the evaporation of the solvents.

"I hope you will give this solution a trial, in order to test its value; and I hope it will prove as useful to you as it has been to me. Yours, respectfully,

"G. F. J. Colburn."

Your reporter has examined the solution above named, and finds the claims of its utility, where time has afforded an opportunity of testing its merits, all that is above demanded; and that it can be rendered serviceable for numerous other medical and surgical purposes. An obstetrician of high merit has already found it a valuable agent in protecting irritable and inflamed nipples of nursing women.

The attention of the scientific was attracted to the article of gutta percha in 1846, and its utility as a plastic agent in the useful arts anticipated.* This substance is the concrete juice of a tree of the order *siphonia*,† which grows on the island of Borneo, and the entire Malayan peninsula. The tree is very large, and bears some resemblance to the *siphonia elastica*, but

* Chemical Gazette, March, 1846, p. 92.

† United States Dispensatory, ed. of 1848, p. 1238.

differs from it in its botanical characteristics. The sap of the tree exudes from its lacerated surface, and quickly becomes solidified on exposure to the atmosphere. It is purified by being boiled in water. At a temperature below boiling water, when, in thin strips or sheets, it becomes as soft and yielding as melted wax or putty, and in this state may be moulded into any form, or stretched out thinner than the finest paper; and when it cools to a temperature below fifty degrees, it becomes tough, and nearly as hard as wood, retaining its plastic shape, not subject to change, except by heat. When in this state, it can with difficulty be cut with a knife or saw, but can be rendered plastic to the hand of a child, by again applying heat, and to any number of times, without perceptibly changing its qualities. Its tenacity is great, a thin slip sustaining a weight of fifty pounds. It burns freely, and emits an odor, when ignited, similar to that of caoutchouc. It is easily dissolved in oil of turpentine, but with difficulty in ether and other solvents of gum elastic.*

The uses of this valuable material in mechanics are numerous, and in the dental and medical profession it can be usefully applied not less frequently. The surgeon may use it in its solid form for bandages, making bougies, catheters, &c. The physician can render it useful for the application of moist bodies to the surface of a patient; and by the dentist, for taking impressions of the mouth, pivoting teeth, and the suppression of hemorrhage.

It would appear that the object to be gained by adding chloroform to a solution of gutta percha, as suggested above by Mr. Colburn, is to hasten the solidification of the solution when applied to a part, by the rapid evaporation of its solvent, as oil of turpentine readily forms a solution from its solid. The action of a solution of this material in suppressing hemorrhage, is practically available only when the small vessels are implicated; and in all cases its action is purely mechanical, by forming a coating over the open mouths of the same. In like manner,

*Eclectic Magazine of Foreign Literature, May No. for 1848, p. 143.

and for like purposes, the "*liquid adhesive* plaster" might be used, which is formed by treating cotton wool with nitric and sulphuric acids, and, when thoroughly washed, afterwards dissolving it in pure sulphuric ether.*

The surgery proper of the mouth, has been too much neglected by the profession in this country, and, without question, to its great injury, by lessening the apparent demand in relation to qualifications and usefulness. But there are a few members in the profession, who have attained a most enviable excellence in the successful performance of the most difficult surgical operations connected with the mouth; and have added some of the most valuable and ingenious assistants, in the form of instruments, which the surgeon can boast.

The success which has attended the practice of Dr. S. P. Hullihen, of Wheeling, Va., and the instruments which he has invented, are worthy of a careful examination by the profession, and particularly those employed in the

### *Treatment of Palatine Deficiencies.*

The uvula often, from numerous causes and diseases, demands excision. The peculiar shape, density and position of this organ, often renders the operation, although simple, one of delay to the operator, and fatigue to the patient, when performed with the knife or scissors, aided by the tenaculum or forceps. These difficulties are wholly obviated, however, by a simple and ingenious instrument, invented during the year 1843, by Dr. S. P. Hullihen,† and known as *uvula scissors*.

This instrument is the common scissors of the surgeon, with flat blades, with the addition of a duplicate pair of blades attached to the handles of the same, on their external surface, when used in the operation; and which, instead of having a cutting edge, are supplied with teeth to retain the parts which they clasp, firmly, when grasped between their jaws. The duplicate blades are so arranged mechanically, that they close a

* American Journal of the Medical Sciences, April No., 1848, p. 577.
† American Journal of Dental Science, vol. vii, p. 274.

line earlier, in advance of the cutting blades which divide the organ, leaving the amputated portion between the duplicate or forcep blades of the instrument. The instrument here shown, will explain itself more readily and fully than any descriptive language.

With this instrument, the operation of excising the uvula is completed without any preparatory steps, and more effectually than with any other instrument known to the examiner. This instrument is also capable of being applied, by increasing the sharpness and length of the teeth of the supplementary blades, and the size of the instrument, for excision of the tonsils. As before shown, the instrument is not a recent invention, but is one that constitutes a "palate set," invented and used by Dr. Hullihen, and in presenting the same to the consideration of the Society, it is thought that the age of this instrument should be no barrier against its introduction in the report; and more particularly since a similar instrument in feature and purpose, was presented in the year 1846, by Dr. Beatty, before the Dublin Surgical Society, and claimed as the invention of Mr. Carte.*

The instrument was invented, and the first pattern constructed by Mr. Kyter, of Wheeling, during the winter of 1843, and afterwards, during July, 1845, the pattern was delivered into the hands of Mr. Wm. R. Goulding, surgical instrument maker, New York City, who since has kept the instrument for sale.† Without entering into an examination of the historical relationship of Mr. Carte's instrument to Dr. Hullihen's, and with the above facts for guides, the credit of invention, in this country, and priority of application of the instrument under consideration, must be awarded to Dr. Hullihen.

*The Curved Forceps.*

This is an instrument invented also by Dr. Hullihen, the use of which is confined to palatine operations. Its use is to hold

* Dublin Medical Press, May 13th, 1846; and Braithwait's Retrospect, part 13, p. 245.

† American Journal of Dental Science, vol. vii, p. 274.

the velum, while the cleft edges are being pared off. Its construction is a slender forceps laterally curved, its joint morticed near the end, the beaks occupying the extreme of the same end or extremity of the instrument, and meeting only at the point, and terminating in fine sharp teeth.

The merits of this instrument consists in the freedom with which it can be used, and the command which the instrument gives the operator over the parts, while employing Hullihen's

### *Velum Bistoury.*

All operators for *staphyloraphy*, from Graffe to the present, have used, for paring or making raw the edge of the cleft, the round pointed bistoury, sometimes with a slight modification of form; or the curved scissors of the surgeon. But all that will contribute to abridge the length of the operation is to be sought, and the means for giving the raw surface or cleft edge a regular and even line, is to be consulted; and to this end, the velum bistoury is peculiarly adapted. The manner in which this instrument acts is simple, however effecting all that its design promises. "After first seizing the cleft edges of the velum at the base of the uvula, with the curved forceps, then putting the velum on a stretch, the bistoury, with its back towards, and against the palate bone, should be pushed through the velum, near its cleft edge. The bistoury, if then opened, after the manner of opening a pair of scissors, which will pare off the edge of the velum in the most even and speedy manner possible." At this stage of the operation, the parts are left ready to receive the ligatures, which may be intruduced by Dr. Hullihen's instrument for the purpose, known as the

### *Acutenaculum.*

The value of an instrument, that should answer all the important demands which the operation imposes on the agent which is to effect the introduction of the ligatures for the completion of the operation, and upon which the entire success of the operation rests, is plainly understood, when we examine and ascertain that hardly an operator of an established character, who has given much attention to this operation, and acquired distinction in the performance of the same, has not felt the deficient merits of the instruments before used for introducing the ligatures for uniting the two edges of the cleft, and have attempted to supply the deficiency by some improvement or invention.

Thus Roux employed, for carrying and introducing the ligatures, needles of a small curve, and a small dressing forceps for a needle-holder. Ebel used short straight needles, and a needle-holder. Donige used a long needle, like an aneurismal needle, with a sudden curve, and a sharp point, with an eye immediately behind it, and the shaft held in a handle bent downwards for convenience. This needle threaded, he proposes should be passed behind, through either edge of the cleft; and when the thread is shown on the inside of the needle's curve, it is to be caught by a tenaculum or forceps, and drawn forwards and completely through; the needle itself, still unthreaded, is thrust through the other edge of the cleft, from behind outwards, and the thread again caught on its appearance, as the short curve of the needle is thrust through, by the tenaculum or forceps, and drawn forwards without the needle's eye, and the needle, being thus set at liberty, is entirely withdrawn, and the thread passed through each other and tied. Werneche used a needle with an eye in front, and a whale-bone handle. Lesenberg's needle was similar to Donige's, but its point could be protected by a guard. Krimer also used a similar needle, which could be closed.* Prof. N. R. Smith, of Baltimore, uses a long lancet-shaped needle, and furnished with a notch for the reception of a ligature, and to be forced through the parts with a forcep-like holder. Dr. J. C. Warren, of Boston, first used the needle of Doniges, but experiencing difficulties from its defects, proposed the use of a curved needle with a movable point, which, after having been passed through the soft palate, can be separated from its holder, unthreaded, and having been refixed and

* Chelius' System of Surgery, South's Translation, Philadelphia ed., 1847, vol. ii, p. 32.

threaded with the previously unemployed end of the ligature, it is passed through the other edge of the palate curtain, and separated from the stem or holder as before. Mr. Lyston introduced his ligatures by the means of a curved needle, fixed in a handle. With this instrument, the ligatures are introduced through the palate curtain, "the noose of which is caught by a blunt hook and pulled through into the mouth." Müller uses a needle half an inch in length, and of a great curve, and mounted on a cylindrical neck, "a part of which is held in the grasp of a port, (*Schwerdt's*,) and the other part, made rough, is intended to be grasped by the forceps of an assistant. The cutting edge of the needle being wider than the diameter of its neck, it will make an opening large enough for the easy transmission of the ligature."*

To all of these instruments practical objections are urged; and all are deficient, by rendering it difficult to introduce the needles uniformly at the proper position in the palate, and the still greater uncertainty of the needles piercing the back part of the palate, as they are pushed through, in such a line as to place the ligature, not only at the proper distance from the margin of the cleft; but also so as to secure strength of the parts, to sustain the approximation of the cleft when the ligatures are put on the stretch, and effect a parallel approach of either side of the cleft, without puckering the edges between the ligatures, and securing a meeting of the back edge, at the same time, of the anterior. These difficulties are experienced by the best operators. Dr. J. Mason Warren, in a recent article on this operation, observes, that "the mucous membrane of the hard palate, which, as often happens, is too much contracted to allow of the manœuvres of the needles and forceps, in carrying the stitch from before backward; I have used the *crochet-aiguille* of Schwerdt. This instrument consists of a double hook, pierced near its point with an eye, having a mechanism like the spring forceps of Assalini."†

* Chelius' System of Surgery, South's Translation, Philadelphia ed., 1847, p. 33.

† American Journal of the Medical Sciences, April No. for 1848, p. 331.

The design of *Hullihen's acutenaculum* is to obviate the above, and other objections connected with the instruments used by surgeons, for the purpose of performing the operation under consideration; and the object of the design has been most fully accomplished in the workings of the instrument; and your report would offer it to the profession, as holding a superiority over all others for the introduction of ligatures in most positions in the operation of *staphyloraphy;* from the command which is given the operator over the instrument or needle, and the parts operated on, together with the uniform precision and facility with which the instrument executes the duty of its office in the hand of an operator.

The instrument is composed of two parts: a staff and a slide. The staff is a small steel bar, six inches in length and an eighth of an inch in breadth, with an arm at its superior end, rising at an obtuse angle from the staff, and half an inch long. On the external or superior side of this arm, a duplicate arm is retained by a spring-temper attachment, which brings the two arms in close contact, forming the jaws of the instrument. Between these two arms, and on the duplicature, is a small groove, formed to receive the ligature; and when the ligature is pressed between the jaws of the instrument, they open, and it slides to the point for its reception, and immediately below which the jaws are perforated with a hole for the introduction of the needle, during its employment in an operation. Two inches from the inferior end of the staff, a pair of rings are affixed, to receive the thumb and index finger—the rings standing parallel with the staff, and sideways to the direction of the arm of the instrument. A slide, formed of steel, equal in length, thickness and breadth to the staff, and made to fit the upper surface of the staff, and to move with ease up and down, by the assistance of guides placed on the same. From the superior end of the staff is a short, straight, spear-shaped needle, constructed with an eye back of its point, with a small notch opening to it from its superior surface. When the ligature has been fitted to its place of reception, and the slide adjusted to the staff, the slide is forced upwardly, and the needle and jaws approach each other, and the needle penetrates the hole in the latter, just under the ligature between the same, and is caught in the notch of the needle, and its eye threaded. When the slide is drawn backwards, and the ligature through the velum, the introduction of the stitch is completed.

The manner in which this instrument places the ligatures in the velum, and its claims, are plainly shown in the following extract from the communication of Dr. Hullihen to the committee of practical dentistry, on the transmission of the same for their examination.

"After first putting a ligature in the notch between the jaws, at the upper end of the instrument, and passing each end of the ligature between the little ears just behind the jaws, the slide upon which the needle is mounted is drawn backwards. Then holding the instrument between the thumb and index finger, the ends of the ligature *hanging over the thumb*, the head or jaws of the instrument is thrust behind the velum; then pushing the slide upwards, the needle passes through the velum, into the hole for its reception in the jaws, just behind where the ligature is confined at this moment; the ends of the ligature, hanging over the thumb, are seized and pulled upon until it slips from the notch where it was first placed, into the eye of the needle. The needle is then withdrawn, and with it the ligature is brought through the velum."

The advantage of this instrument over curved needles, or any other instruments now in use for placing ligatures in the velum, is this: it enables the operator to hold the velum with the point of the needle, as with a tenaculum, until he satisfies himself where the stitch should be introduced. By this means, the stitches may be always inserted the proper distance from the edge of the velum, and on both sides directly opposite each other; a matter of high importance in the successful treatment of cleft palate. I have operated on eleven cases for cleft palate with this instrument, and one case was only nine years of age, and in every case with success."

*Early Operations for Hare-lip and Cleft Palate,*

Have been proposed by high authority. In 1843, it was advocated by Dr. J. Mason Warren,* and more recently he has asserted that his further experience has supported the position then assumed, and the truth of the arguments then offered, as inducements for the early performance of the operation for palatine fissure in connection with hare-lip.† But the method proposed by Dr. Hullihen, for operating at an early age for the remedy of both of the defects named, is not only original, but more simple in its action, and effectual, than any other surgical means hitherto adopted. In the communication from Dr. Hullihen, previously quoted, he observes:

"I have another very simple contrivance to submit to you, that is connected with the treatment of deficiency of the palate; one I value more than all the rest, because I can accomplish more and better results with it than with all the rest together. It is composed of two pieces of common adhesive plaster, cut so as to fit the cheeks of an infant. To this is a narrow slip of gum elastic, cut so as to fit over the upper lip and close to the nose. It is used for closing a cleft in the alveolar arch. With this simple contrivance, I can close the widest cleft in a week or ten days, in any infant under three months old. For a full description of the management of such cases, I beg to refer you to my paper on hare-lip, in the fourth volume of the Dental Journal. Since the publication of that paper, I have employed the gum elastic, as just described, and have likewise united the cleft edges of the alveolar arch, by paring off the the ends as soon as they touch each other, and then drawing them together with a slip of gum elastic, more powerful than would be proper to use at any former stage of the treatment, and thus retain them until united."

To the above it is not thought necessary to add any thing more than to observe, that the cartilaginous state of the bones in early infancy, requires but little force to bring them into any

* New England Quarterly Review of Medicine and Surgery, for April, 1843.
† American Journal of the Medical Sciences, for April, 1848, p. 337.

desired position. But in removing the deformity of a cleft alveolar arch, a force of a two-fold nature is often required, both to bring the edges together, and at the same time compress any projection of the alveolar process which may exist. In the proper application of the adhesive straps, may be found this happy combination."

The fixture, after being prepared, can be applied in the following manner: After the adhesive surface has been properly warmed, or evenly touched with a *very thin* coating of oil of turpentine, one end should be quickly applied and attached to the cheek of one side; the cheeks of both sides should then be pressed forwards by the palms of the hand of an assistant, when the strap should be passed over the lip, and be attached to the cheek of the opposite side, thus confining the cheeks forwards, and exerting upon the jaw sufficient force to close, in a few weeks, the widest cleft of the alveolar arch, or approximate a palatine fissure. The time required for completing an operation, is governed more by the age of the child than the width of the fissure.*

In the department of mechanical dentistry, the improvements have been constant and rapid. At the same time that mechanical excellence of completion has been anxiously sought by the accomplished and educated portion of the profession, it has not been at the expense of physiological and pathological attainments, or surgical adaptation. In that division known as

### *Pivoting an Artificial Crown on a Natural Fang,*

There has been as marked a change in the practice of the profession, as in any of its departments; and that from the increase of knowledge of the functions of the system during health and disease. But still the operation is much too frequently resorted to, when the indications forbid it. If inflammation has destroyed the nervous pulp in the fang of the tooth, it may be laid down as a sufficient evidence that the parts will suffer disease from the retention of the fang. And even under certain

* American Journal of Dental Science, vol. iv, p. 244.

circumstances of local or constitutional disease, when the nervous pulp has not suffered inflammation, a failure could be plainly foretold, from the inflammation which would follow the operation. At the best, the operation is only a temporary one, as the fang, sooner or later, becomes offensive, and acts as an irritant to the surrounding parts, the fang having taken on some one of the forms of disease named, when considering the destruction of the nervous pulp, preparatory to filling the fang of a tooth.

When a patient, having healthy organs, of adult age, the density of whose teeth is great, and whose soft tissues are firm in structure and healthy in their action, consults a dentist, for the purpose of supplying the loss of a natural crown of a tooth, by engrafting an artificial one on its fang; if the local indications are favorable, it becomes a case offering the least objection to the operation. For the better execution of the operation, G. F. J. Colburn, dentist, of Newark, N. J., has constructed and offered to the use of the profession what he calls a

### *Pivot Guage.**

The instrument is simply a piece of ivory, two inches long, half an inch wide, and one-fourth of an inch thick, pierced with a number of holes of different sizes, from the smallest to the largest pivot, which is "bushed" with silver. The instrument is applied to a useful purpose, in the following manner: After having chosen a drill to enlarge the natural canal in the fang, of a size to fit some one of the holes, a pivot is made and forced through the hole corresponding to the size of the drill, thus not only compressing the fibres of the material, but securing equal dimensions of the pivot, permitting it to fit the place of reception, without exerting force in its adaptation, not only to the artificial crown, but what is of more importance, to the fang. It is well known, that by exerting physical force in introducing a badly shaped pivot—one of unequal dimensions, that inflam-

* Some members of the Society assert that this instrument has been known to them as having been used in practice for some years.

mation is often lighted up in the peridental membrane lining the socket of the fang, which results in the necrosis of the same.

The gentleman above named has also suggested, in his communication previously presented in this report, the use of gutta percha, to protect not only the exposed end of the fang, but render the junction of the fang and artificial crown more complete. The value of the article named above, for the purposes in question, your report cannot offer any opinion based on fact, other than those accompanying Mr. Colburn's communication, as sufficient time has not intervened to test experiments. But, arguing from analogy, it is thought that the junction of the fang and artificial crown can be rendered, by this means, permanently more complete, even after having been fitted by the use of the male and female files used for this purpose by Dr. E. Townsend of Philadelphia,* than by any means in use by the profession.

The means and material employed in

*Taking Impressions,*

Have received some recent and important additions. G. F. J. Colburn has added a duplicate rim to the ordinary wax-holder, which subserves the purpose of protecting the wax impression from injury in its withdrawal, and also may be rendered useful in more speedily solidifying the material, if it be wax or gutta percha, in which the impression is taken, while in the mouth, by filling the chamber between the two rims of the holder with cold water.

The use of gutta percha for taking impressions of the mouth, for obtaining models or casts of the same, was made known to the profession by the publication of two papers in the Dental Journal; one by G. F. J. Colburn, of Newark, N. J.,† and the other by W. P. Blake, New York City.‡ That this agent possesses considerable superiority over wax, in obtaining a perfect

* Harris' Principles and Practice of Dental Surgery, Phila., 1848, p. 612.
† American Journal of Dental Science, vol. viii, p. 258.
‡ Ibid., vol. viii, p. 278.

impression, is evident. It also gives an even surface to the plaster cast, that is not obtained from impressions taken in wax. This material is subject, however, to practical objections under certain circumstances and peculiarities of case.

*Double sets* of artificial teeth sometimes demand, for their firm retention, some expansive force, which shall cause the parts of the artificial piece, to occupy firmly their position on the alveolar border during all positions of the jaw. For this purpose, spiral springs, attached to each side of a double artificial denture, uniting by this means the superior with the inferior plate. To the ordinary spiral spring, Dr. S. P. Hullihen has made an improvement, which he has been using in his practice for some years, and which your report would draw the attention of the association to its consideration.

The spring consists of two long arms, formed of gold plate at each end, and these united in the middle by the ordinary spiral spring, which is from one-fourth to half an inch in length.

This bit of spiral spring acts as the power, when the opposite ends of the arms are made to approach each other. At the other end of the arm, the point of union, thus making the arms compound lever of the third class.

This spring embraces all the expansive power of the ordinary spiral spring in use by the profession, with the advantage of obviating a lateral curvature, which not only tends to the displacement of the plate, and unequal pressure on their irritable base, but oftentimes troublesome to the buccal surface of the cheeks. The spiral spring of Dr. Hullihen demands that the point of attachment to the two plates be exactly opposite each other, for should the exactness of mechanism in this particular be violated, the power would be thrown unequally on the arms, producing evils that can only be avoided by remedying the original imperfection. But in old persons, where the angle of the inferior jaw has become obtuse, and the occlusion of the jaws irregular and tremulous, the difficulty becomes one not so easily corrected.

### *Atmospheric Pressure.*

This principle of nature has been taken advantage of for the purpose of confining gold plates in the mouth, on which are mounted artificial teeth.

The principle on which the teeth are retained is well explained by the simple experiment of pressing a circular piece of wet leather against an even surface of a flat stone or tile. If the air is all forced from between the leather and the solid body, and a cord be attached to the centre of the leather, and its texture is sufficient to prevent the passage of air through the same, and stiff enough not to be puckered or drawn together; there can be exerted a force of as many times fifteen pounds, as there are square inches of surface covered by the leather, before detaching it.*

In like manner, if a correct impression, and dies or casts are obtained of an alveolar ridge, and a metallic plate be accurately fitted to the same by the means of the dies, so as to exclude *all* air from between the surface it covers and the plate, it will be retained in connection with the parts, with a great deal of firmness. But it is seldom that all steps in the construction of a piece of dental mechanism has been rendered so complete as to retain a firm adhesion of the plate to its alveolar base by atmospheric pressure; a stratum of air is permitted to intervene between the plate and the parts on which it rests. But notwithstanding which, the artificial plate is retained in position by the partial success of the principle proposed, and by the assistance of the same force which holds together the particles of a solid body, and which acts in holding together the heterogeneous particles of two bodies, which are already separated, provided they are brought within sufficiently intimate contact.†

Otto Von Guericke first made the remarkable experiment with the *Magdeburg hemispheres*, which are two half globes of

*Arnott's Elements of Physics; Philadelphia ed., 1831; vol. i, p. 182.
†Müller's Principles of Physics and Meteorology, Philadelphia ed., 1848.

metal, and are fitted to each other, so that the lips, when touching, may be air-tight; and which consisted in making a vacuum in the hollow between the two *hemispheres*, by exhausting the air from the same.* This experimenter, with *hemispheres* a foot in diameter, once on a public exhibition, after exhausting the air from the chamber of the same, attached six coach horses of the emperor to the *hemispheres*, which were unable to pull them asunder.

When, from the horizontal conformation of the palatine vault, &c., has rendered it difficult to adapt and sustain a plate for mounting artificial teeth, on the principle before named, it has frequently been the practice of many of the profession, to construct a chamber between the plate and the palatine arch, which shall enable a more perfect vacuum to be formed than can be attained by any other principle than that of the experiment just referred to. The method resorted to for attaining this result, is this: after having procured a correct impression of the mouth, and a plaster cast, let a small addition of material be made on that part of the cast that corresponds to the roof of the palate vault, of the thickness of a worn shilling coin, taking care that it is not of such dimensions as shall change the original character or shape of the cast, at such parts as the edge of the plate, in the posterior portion of the palate, is to meet, as the "fit" of the plate on its margin must be air-tight, or the design of the operation, so far as the relation of the plate is implicated, is violated. After the above directions have been complied with, the cast is prepared for obtaining the dies or metal casts, which are procured in the usual way.

At the time Guericke performed his first experiments, there being no air-pump, he emptied the cavity in the hemispheres of air, by first filling them with water, and then extracting the fluid by a common syringe applied at the bottom.† A like method is adopted, when the plan last described is used for introducing an artificial piece of dental mechanism by atmospheric pressure. When the gold plates are properly adapted to

* Müller's Principles of Physics and Meteorology, Philada. ed., 1848, p. 137.
† Arnott's Elements of Physics, Philada. ed., 1831, vol. i, p. 282.

the palate roof, and ready for a trial to determine the permanence of its retention, the plate is filled with water, and then pressed to its position, and retained until the tongue and buccal muscles, aided by inhalation, fulfils the office of a pump, and extracts the water, leaving an exhausted vacuum, which, if perfect in mechanical construction and adaptation, will sustain the artificial substitute with firmness.

To this method of retaining plates there are some weighty objections. When the operation is proposed on young subjects, or when a predisposition to inflammation is developed in the patient, or the construction of the plate is such as to institute congestion of the vessels of the soft tissue, where the edge of the plate meets the roof of the palatine arch; a pathological change is liable to be instituted; and if the irritating cause be long continued, a fungous growth may fill the vacuum or chamber intervening between the plate and palatine vault, from a long continued vascular injection of the parts; giving rise not only to a troublesome local disease, but destroys the adhesion of the plate to the parts, and renders the piece of dental mechanism useless.

The report, then, would offer the opinion, that on physiological and pathological grounds, the *indiscriminate use* of this method of inserting whole or parts of sets of artificial teeth is inadmissible in general practice. And that the most favorable cases for application of the principle are, where it is most demanded; namely, in entire upper sets, the patient advanced in age, when the soft tissues have lost the vascularity of early life, and the teeth have *long* been extracted, and the alveolar processes not subject to a further waste or change, and the parts indurated. And even when this principle is resorted to in favorable cases, it is important that preventive measures be resorted to, to prevent the objections that might follow the use of the principle, by relieving the vessels, by the use of friction daily with the finger on the soft parts covered by the plate.

### *Artificial Palates and Velums*

Are demanded, when the functions of these parts are lost by their absence, either from disease or formation, to retain the air, after its introduction into the mouth from the lungs, until the letter-type of articulation is impressed; and the artificial means that shall subserve this purpose to the greatest extent, when a lesion exists in these organs, will be most cordially embraced by the profession.

There has ever existed a serious difficulty in all mechanical contrivances for supplying the loss of the velum, both from the immobility of the material of which the artificial is composed, and the consequent troublesome inflammation of the small muscles which come in contact with the same; and the difficulty of constructing a piece of mechanism which would permit the proper volume of air, and at the proper period, to pass to and from the mouth, by the nares.

Your reporter presents a model of an artificial palate, designed by Dr. S. P. Hullihen, which did obviate the difficulties above named, and fulfilled the design of its construction, in the case for which it was made.

In the communication accompanying the model, Dr. Hullihen observes, "In cases where the *velum has been destroyed* by mercury, syphilis or other causes; an artificial substitute may be required. Herewith I send you a model of one I constructed about twelve years since, which has answered a most excellent purpose. The peculiarities of this contrivance are, first, a valve made to fit the posterior opening of the nares; secondly, the attachment of this valve to a slider, by which the patient is enabled to adjust the valve while in the mouth, in such a way as to admit through the nares just the quantity of air desired. Thirdly, the mounting of the valve on a spiral spring, which vibrates backwards and forwards, as the breath is inhaled or exhaled, thereby answering, to a great extent, the purpose of a velum."

In conclusion, your report would offer it as its opinion, that the advance of the profession, both in its acquirements and appreciation by the community, has been most marked during the last few years, and even since the last meeting of this association. A profession is estimated by the same natural law which leads the mind to the discovery and appreciation of beauty; namely, the associations connected with it. If the practice of the dental profession demands science and education for its study, and its dispensation be associated with human life and human happiness and comfort, then must it stand in the position of a calling of high estimation. And that such is gradually becoming the case, may be perceived, from the well marked division which is widening between the regular and irregular practitioner; and the value which is placed on the labors of a member of the profession, in whom the community have confidence; and the occasional requests to the faculties of dental institutions, for graduates of professional and moral excellence, to fill vacancies in practice.

To be confident that this feature of change is to go on and be permanent, we have only to know that it rests on public necessity. Go where we may, we find the community jealous of the qualifications of the profession, and loud in complaint of its abusive practice.

The members of this association, then, are under the strongest ethical obligations, not only to preserve the character of this change, but to hasten it in its progress, by every effort of labor and contribution that may tend to distinguish the dental profession for general and extensive kowledge, and dignity of sentiment, and prompt effusion of beneficence.

And finally, the report, in marking the progress of the profession during the year for which it is prepared, can say, that the ceaseless and unwearied exertions of master-minds of the profession, have been constantly urging on improvements in the various departments of the profession; and have elaborated a work, which will require years of toilsome study justly to appreciate; and while we are far from indulging the Utopian idea of the "millenium" of the profession, still the battle-axe of science and industry has struck the shield of empiricism and ignorance, until the resonance of its blows would appear as if driven by the hands of giants; and the light of true philosophy is at last breaking in upon the very complex and difficult subjects of dental inquiry; and where formerly darkness and positive uncertainty existed, and keen penetration beheld only confusion, even common minds now begin to see clearly divisions and beautiful arrangements.

All of which is respectfully submitted.

